# Wound Myiasis Caused by* Sarcophaga (Liopygia) Argyrostoma* (Robineau-Desvoidy) (Diptera: Sarcophagidae): Additional Evidences of the Morphological Identification Dilemma and Molecular Investigation

**DOI:** 10.1155/2017/9064531

**Published:** 2017-01-29

**Authors:** Annunziata Giangaspero, Marianna Marangi, Antonio Balotta, Claudio Venturelli, Krzysztof Szpila, Antonella Di Palma

**Affiliations:** ^1^Dipartimento di Scienze Agrarie, degli Alimenti e dell'Ambiente, Università degli Studi di Foggia, Via Napoli 25, Foggia, Italy; ^2^Unità Operativa di Geriatria, Ospedale M. Bufalini, AUSL della Romagna, Ravenna, Italy; ^3^Dipartimento di Sanità Pubblica, AUSL della Romagna, Ravenna, Italy; ^4^Chair of Ecology and Biogeography, Faculty of Biology and Environmental Protection, Nicolaus Copernicus University, Lwowska 1, 87-100 Toruń, Poland

## Abstract

In Mediterranean countries,* Sarcophaga (Liopygia) crassipalpis*,* Sarcophaga (L.) argyrostoma, *and* Sarcophaga (L.) cultellata* share the same ecological niche and can be responsible of myiasis. In this study, the main morphological characters of a larva found in a hospitalized woman were described and illustrated by light and SEM microscopy and the features discussed. Then, a fragment within the mitochondrial encoded cytochrome c oxidase subunit I* (coxI)* gene of ~735 bp was amplified and sequenced. The molecular investigation was necessary to confirm the species* Sarcophaga (Liopygia) argyrostoma* (99% of identity). Our findings showed that morphological descriptions of larvae of three Mediterranean species of* Liopygia* available in several papers might not be clear enough to allow for comparison and correct identification. Until results of reliable comparative studies of larvae of all three species will be available, the use of molecular tools is crucial, to avoid misleading or incomplete identification, and in particular when a myiasis becomes a legal issue.

## 1. Introduction

In Europe, 33* Sarcophaga* Meigen species attracted to large carrion have been described [1]. Several of them pose confirmed status of facultative myiasis agents [2]. Among them, in the Mediterranean countries, three* Sarcophaga *species belonging to the same subgenus* (Liopygia)* share the same ecological niche:* Sarcophaga (Liopygia) crassipalpis* Macquart, 1839*, Sarcophaga (L.) argyrostoma *Robineau-Desvoidy, 1830, and* Sarcophaga (L.) cultellata* Pandelle, 1986 [3]. These species are included in the category of “nosocomial myiasis agents” because they have been found in wound myiasis affecting hospital patients who are immobilized, often old, and poorly cared [4–8]. Flies are attracted by the smell of fresh or infected and untreated wounds and lay eggs (or larvae as in the case of* Sarcophaga*), which then develop in necrotic or injured tissues and sometimes cause or exacerbate a wound [9]. This can often become a legal problem, which entails an urgent need to identify the larvae.

Where the geographical species distribution overlaps, as happens for these three* Sarcophaga* species in the Mediterranean area [3], and only one single larva is available, differentiation of these three species becomes very challenging [3, 10]. Thus, cross-checking of the morphological and molecular data is highly recommended for both adult [11] and larval specimens [10].

The mitochondrial encoded cytochrome c oxidase subunit I* (coxI)* gene has been shown to be a major candidate gene for molecular identification of forensic flies [12] and is currently the most widely used genetic marker for differentiation of* Sarcophaga* species of forensic interest thanks to its intraspecific (below 1.5%) and the interspecific divergence (2–2.5%) [13, 14].

The aim of this contribution is to report a case of nosocomial myiasis, to describe and illustrate the main morphological characters used in identification of the collected larva, compared to those available in the literature, and to highlight the need for molecular support for correct identification.

## 2. Materials and Methods

In September 2014, a 101-year-old female patient who had been bedridden for months in a geriatric home in Cesena was specifically admitted to Bufalini Hospital with a “parasitic infestation” of a wound. A single larva was found on the wound, removed with pincers, and preserved in ethanol (ETOH 80% v/v). The wound was then disinfected using chlorhexidine.

The larva collected was subsequently sent for identification to the Parasitology Unit of the University of Foggia. Here it was cut into three parts; thoracic and terminal segments were subjected to microscopy, while the abdominal segment was used for molecular identification.

### 2.1. Microscopy

The larva portions were first macerated in lactophenol for one week at 45°C on a hot plate and then mounted on a Hoyer's-embedded slide. Observations, identification and light images were obtained using an Olympus BX51 light microscope (LM) equipped with an Olympus E330 camera.

The larva portions were then remounted from the slide and prepared for scanning electron microscopy (SEM). Hoyer's fluid was removed with water, and the larva portions were dehydrated using a graded ethanol series, dried with a Leica EM CPD300 critical point dryer, mounted on SEM stubs using conductive carbon adhesive tabs, and sputter-coated using an Edwards S150A sputter coater apparatus. A Hitachi TM3030 tabletop scanning electron microscope was used for observations and micrographs.

The III instar larva was identified using the key for myiasis-causing flies [15] and for the Sarcophagidae family to subgenus level [10]. The most distinctive characters available in literature for* Sarcophaga (Liopygia)* spp. identification, that is,* Sarcophaga argyrostoma *[8, 10, 16, 17],* Sarcophaga crassipalpis* [18], and* Sarcophaga cultellata* [3], were considered. In particular, the presence/absence of spines/warts on the t2 interband area, the number of lobes on the anterior spiracles, the shape of the posterior spiracles, and cephalo-skeleton were compared (Table 1).

### 2.2. Molecular Investigation

DNA was extracted using the Nucleospin Tissue kit (Macherey-Nagel, Netherlands) according to the manufacturer's instructions. The extracted DNA was eluted in 50 *μ*L of distilled water and the sample was stored at −20°C.

Universal primer pairs (F1/R1; F2/R2; F3/R3) (Table 2) [20] were used to amplify fragments of different sizes within the cytochrome oxidase subunit I gene. The PCR reaction mix contained 5x Phire Reaction Buffer (Thermo Scientific, USA), 200 *μ*M of dNTPs (Qiagen, USA), 10 *μ*L of each primer pairs, 1U Phire Hot Start II DNA Polymerase (Thermo Scientific, USA), and 1 *μ*L of genomic DNA in a final volume of 20 *μ*L. The PCR protocol was as follows: denaturation at 98°C for 30 s, followed by 98°C for 5 s and 59°C for 30 s for 35 cycles, and finally 72°C for 1 min. A negative control (PCR water) was included in each PCR run. The PCR products were run on 1.2% agarose gel and positive samples were purified with Exonuclease I (EXO I) and Thermosensitive Alkaline Phosphatase (FAST AP) (Fermentas) enzymes.

Amplicons obtained were directly sequenced in both directions using the ABI PRIMS BygDye Terminator v. 3.1 Cycle Sequencing Kit (Applied Biosystems, Foster City, California, USA) with the same primers as the respective PCRs reaction, according to the manufacturer's instructions. Sequences obtained were determined on an ABI PRISM 3130 Genetic Analyzer (Applied Biosystems), electropherograms were inspected by eye, and consensus sequence was determined assembling different PCR fragments.

Our sequence was aligned with the available nucleotide sequences in publicly databases, using the BLASTn software and the Muscle application implemented in SeaView 4. Moreover, our sequence was aligned with all the* S. argyrostoma* DNA barcode sequences [ID numbers: NICC0085, accession number: JQ582081; NICC0637, accession number: JQ582123] [21] using Bioedit V.5 software and the Muscle application implemented in SeaView 4. Phylogenetic analyses were conducted in MEGA6 using the Neighbor-Joining method. The evolutionary distances were computed using the p-distance method and were in the units of the number of base differences per site. The analysis involved 45 nucleotide sequences. Codon positions included were 1st + 2nd + 3rd + Noncoding. All positions containing gaps and missing data were eliminated. There were 61 positions in the final dataset. The tree was rooted using a* Wohlfahrtia magnifica* sequence as an outgroup (accession number: FJ379616).

## 3. Results

### 3.1. Microscopy

**Figure 1:**
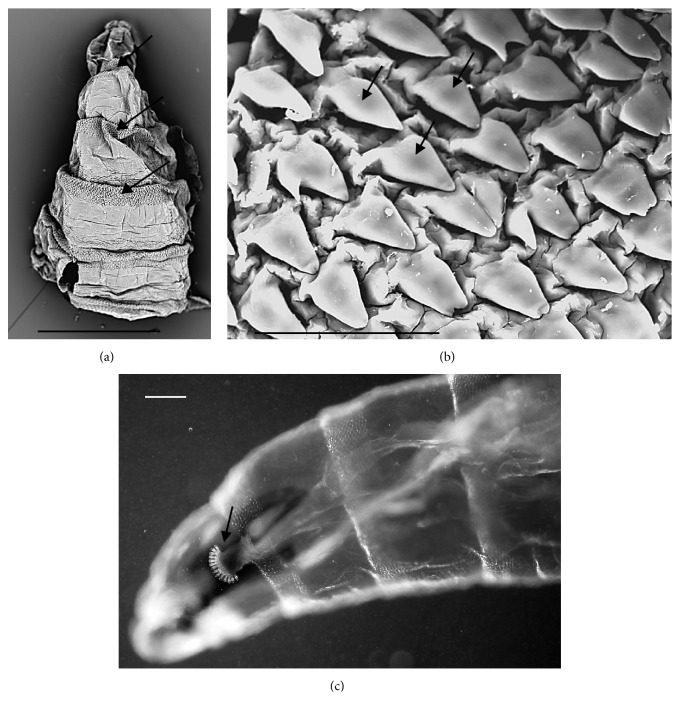
*Sarcophaga *sp. third instar larva. (a) SEM image, anterior dorsal view of the body showing the interband areas free of any spines/warts and the anterior spinose band (arrows). (b) SEM image, detail of the spines on the anterior spinose band areas (arrows). (c) LM image, lateral view of anterior body showing anterior spiracle with 14-digit-like lobes clearly visible. Scale bar: 1 mm (a); 50 *μ*m (b); 100 *μ*m (c).

**Figure 2:**
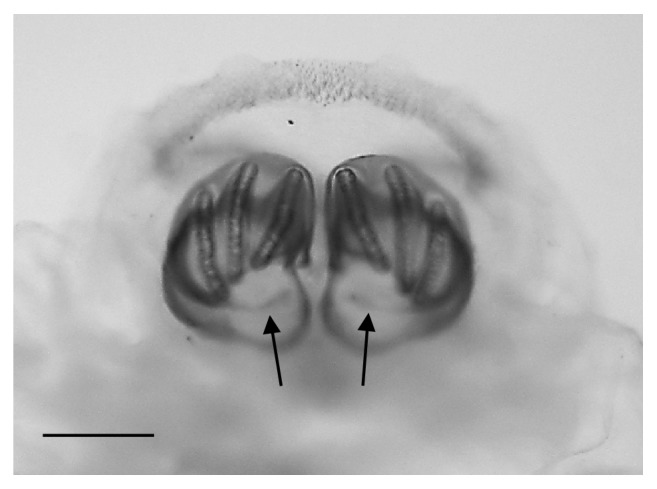
*Sarcophaga* spp. third instar larva: LM image, overview of kidney-shaped posterior spiracles with ventral arches (arrows) not reaching the inner arch area. Scale bar: 100 *μ*m.

**Figure 3:**
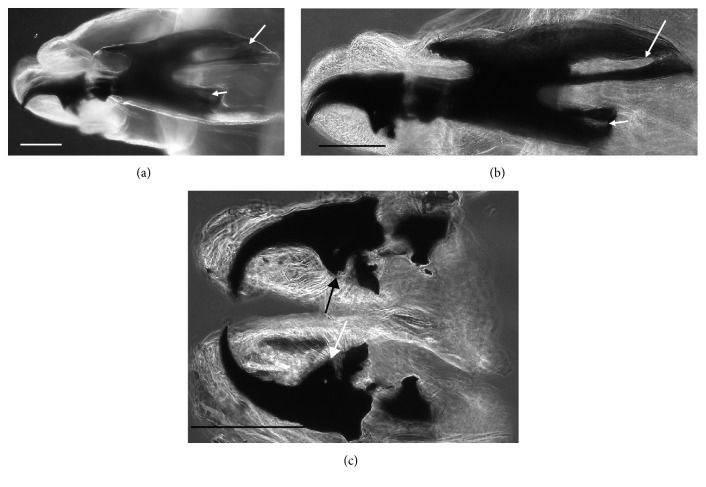
*Sarcophaga* third instar larva: LM image: (a) cephaloskeleton with dorsal cornua showing window apparently 8-shaped (arrow) while the ventral cornua window is smaller but still well developed (arrowhead). (b) Same sample showing a different shape (more elongated and not 8-shaped) of the window due to a different orientation of the sample in the slide. (c) Detail of the ventral bridge of the intermediate sclerite with truncated tip (arrows). Scale bar: 100 *μ*m.

**Figure 4:**
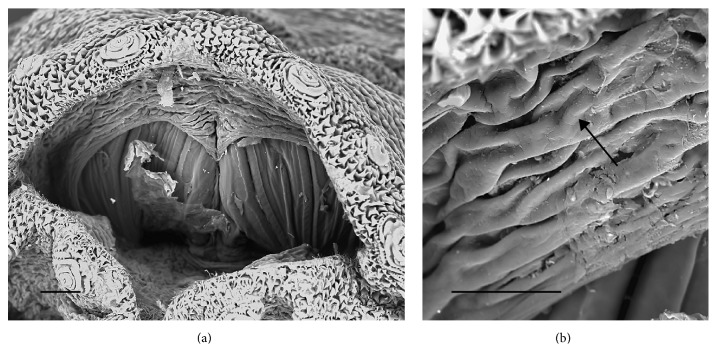
*Sarcophaga *sp. third instar larva: SEM images. (a) Overview of the posterior spiracle cavity. (b) Detail showing highly wrinkled edge of the posterior cavity (arrow). Scale bar: 100 *μ*m (a); 30 *μ*m (b).

The larva examined in this study had the following characters:

1. No spines on t2 interband area (Figures 1(a) and 1(b))
2. Anterior spiracles with 14 lobes (Figure 1(c))
3. Kidney-shaped posterior spiracles (Figure 2)
4. Short ventral arch of the posterior spiracle, not reaching the inner arch area (Figure 2)
5. Ventral cornua of the cephaloskeleton with developed window (Figure 3(a))
6. Dorsal cornua not clearly 8-shaped (Figure 3(a)) and tip shape of the intermediate sclerite apparently truncated (Figure 3(c))
7. Spiracular cavity with very wrinkled edge (Figure 4)

According to the comparative table and illustrations (Table 1), of the seven features above, one (number 5) and apparently another (number 6) point to* S. argyrostoma* species; one character (number 1) points to all three species (*S. argyrostoma* and/or* S. cultellata* and/or* S. crassipalpis*); one character could be related to both* S. argyrostoma* and/or* S. cultellata* (number 3), one to both* S. crassipalpis* and/or* S. cultellata* species (number 4), and two features (nos. 2 and 7) are apparently not associated with any of the three species.

### 3.2. Molecular Investigations

**Figure 5:**
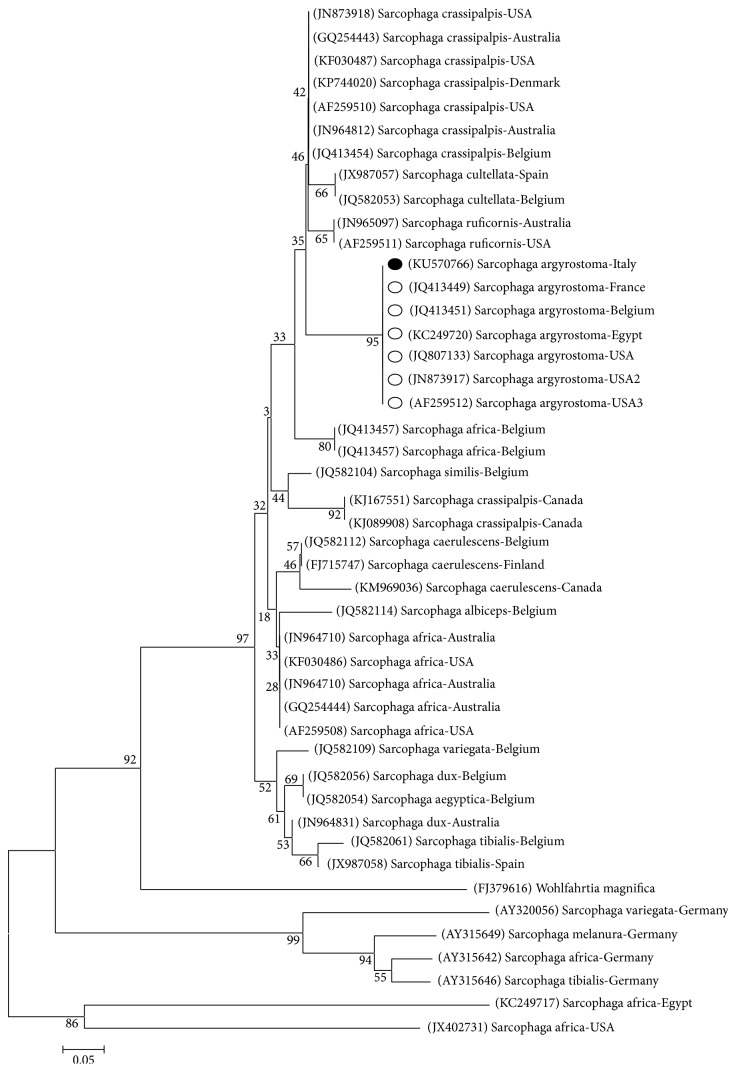
The Neighbor-Joining (NJ) phylogenetic tree based on analysis of the partial cytochrome *c* oxidase subunit I gene of* Sarcophaga *species of forensic interest. The* Sarcophaga argyrostoma* sequence from the present study (black spot), 11 sequences representing the other* Sarcophaga* species belonging to* Liopygia* subgenus, that is,* S. crassipalpis* (9) and* S. cultellata *(2) and 20 other* Sarcophaga* species from GenBank were included in the analysis for comparative purposes.* Wohlfahrtia magnifica *was used as an outgroup. GenBank accession numbers (in brackets),* Sarcophaga* species, and country of origin are reported.

Sequencing provided a good quality sequence of about 735 bp. BLASTn analysis (https://blast.ncbi.nlm.nih.gov/Blast.cgi) showed that our sequence matched the target GenBank* coxI* sequence* S. argyrostoma* with a 99% of identity. Ninety-nine percent of identity was also obtained by alignment with* coxI* DNA barcode region sequence [21]. The sequence was deposited in GenBank under accession number KU570766. The phylogenetic analysis showed that the Italian sequence clusters with the other* S. argyrostoma* sequences (from Belgium, France, Egypt, and USA) in a monophyletic group with a high bootstrap value (>95) and that this clade is distant from the other* Sarcophaga *species (Figure 5).

## 4. Discussion

In this study, the single III instar larva showed some characters that are typical for* S. argyrostoma*, whereas some other characters were not. Thus, according to the morphological characters it was only possible to assume the identification of the single III instar larva as* Sarcophaga (Liopygia) argyrostoma* but a molecular investigation analysis was needed for confirmation.

In addition to* Sarcophaga (Liopygia) argyrostoma*, two other species in the same subgenus are found in Mediterranean countries:* S.* (*L.*)* crassipalpis* Macquart, 1839, and* S. *(*L.*)* cultellata* Pandelle, 1986 [3, 10]. These three species have been recorded either in case reports of wound myiasis in live humans or in supporting forensic investigations of human corpses. In Southern Italy,* S. crassipalpis* was identified in a corpse [18], while* S. cultellata* has been identified in human corpses in Spain [3, 22] and also in Northern Italy [23].

The main key morphological characters for differentiation of the III larval stage in these three species of the subgenus* Liopygia* involve dorsal surface of the II thoracic segment, anterior spiracle digits/papillae, posterior spiracles, and the shape of the cephaloskeleton [3, 10]. In our case, those characters do not appear clearly unequivocal: some key features seem related to all three species, that is, the second thoracic segment interband area [10], and shape of the posterior spiracles [3, 17, 18], while some characteristics cannot be ascribed to any of these three species, that is, the number of lobes on anterior spiracles and edge of the posterior spiracular cavity [3, 17] (Table 1). Moreover, some characters are difficult to read, since their appearance and shape may change with the position of the sample on the slide. An obvious example is provided by the shape of the dorsal cornua window, which appears different on the same sample, merely because of its position (please compare Figures 3(a) and 3(b)). Generally, the main obstacle in the use of morphological markers for identification of larvae of subgenus* Liopygia* is lack of solid comparative analysis. Studies of potentially useful characters, based on a long series of individuals and unified methodology, should solve this problematic situation as it was done for larval stages of some other difficult taxa (e.g., [24, 25]).

Hence, when larvae come from the* Liopygia* distribution area, that is, the Mediterranean countries, the available morphological features may not be clear enough for comparison and correct identification. This problem, coupled with the difficulty of checking all characters when a single specimen is provided, calls for the use of molecular tools.

The robustness of the* coxI* gene as a diagnostic marker to lay the groundwork for identification of these three species of* Sarcophaga *has been demonstrated [21, 26].* Sarcophaga argyrostoma* has been identified using a* coxI* sequence in an elderly patient with an external ophthalmomyiasis (with 97% identity) [6] and very recently in a tracheotomized child's surgical wound (with an identity of 100%) [8]. In the present study, the involvement of* S. argyrostoma* in the wound myiasis is confirmed with 99% identity.

This is the third case in Europe [4, 8]. Cases of myiasis caused by* Sarcophaga* species are apparently few, but it must be underlined that cases of myiasis often remain unrecorded or that, more frequently, identification is partial, that is, at genus level [27–31].

## 5. Conclusion

In conclusion, it is very important to record and correctly identify the species involved in nosocomial myiasis, particularly if patients are moved from geriatric homes to hospital for such problem and thus even become a legal issue.

Correct identification of the myiasis agent provides more detailed information on the responsibility that health units (hospitals, geriatric homes, etc.) must have towards patients. In this view, hygiene, protection from flies by physical barriers (screens or closed windows), efficient waste disposal measures to reduce the smell of decomposition, and insecticide sprays are basic prevention measures that hospitals should take.

## Figures and Tables

**Table 1:** Third larval stage of the most common *Sarcophaga (Liopygia)* species: comparison of the main morphological features, and related iconographic documentation, available in the literature.

Anatomical sites	*Sarcophaga (Liopygia)* species			Our findings
(III stage larvae)	*Sarcophaga argyrostoma*	*Sarcophaga crassipalpis*	*Sarcophaga cultellata*	(LM and SEM)
1st and 2nd thoracic segment	At least anterior part of second thoracic segment without spines/warts on interband area [1, 10]	NONE	Shape of the anterior spinose band of the first thoracic segment [3]	Figures 1(a) and 1(b)

Anterior spiracles	Typal digit-like lobes with 10-11 lobes [17]	Typal digit-like lobes with 12 lobes [19]	Typal digit-like lobes with 15–18 lobes [3]	Figure 1(c)

Posterior spiracles	Posterior spiracle, ovate or kidney-shaped Ventral arch of the posterior spiracle, long, reaching the inner arch area [17]	Posterior spiracle, round Ventral arch of the posterior spiracle, short, not reaching the inner arch area [19]	Posterior spiracle, kidney-shaped A thick well sclerotized peritreme which is incomplete and divided into four regions: (a) a straight inner arch with a triangular protuberance at the ventral tip, (b) a scalloped shaped dorsal arch, (c) a round outer arch, (d) and a short and straight ventral arch. The inner arch is slightly oblique with respect to the sagittal plane of the larva, and the line drawn by this arch is reached by the free tip of ventral arch Inner arch of posterior spiracle oblique, almost parallel to the inner respiratory slit [3]	Figure 2

Cephaloskeleton	Ventral cornua have a developed window Ventral bridge of the intermediate sclerite with the tip truncated [17]	Ventral bridge of the intermediate sclerite with the tip rounded [19]	Dorsal edge of the mouth hooks, including approximately two-thirds of the length of the tooth, being rectilinear and only curved at the tip The parastomal bar is fused to the basal sclerite, and the anterior tip is usually pointing upward (i) The window of the dorsal cornu is clearly 8-shaped (ii) The dorsal bridge is more slender (iii) The window of the ventral cornua is smaller [3]	Figures 3(a), 3(b), and 3(c)

Spiracular cavity	Edge of spiracular cavity, highly scaly [17]	None	Edge of spiracular cavity slightly covered by filamentous spines [3]	Figure 4

**Table 2:** Universal primer sequences within the mitochondrial cytochrome oxidase gene subunit I (*coxI*) (by Kim et al., [20]).

Name	Sequence	Binding site
F1	CCTTTAGAATTGCAGTCTAATGTCA	tRNA-cysteine
F2	GGAGGATTTGGAAATTGATTAGTTCC	220–245 on COI
F3	CTGCTACTTTATGAGCTTTAGG	2–23 on COI
R1	CCTAAATTTGCTCATGTTGACA	1000–1022 on COI
R2	CAAGTTGTGTAAGCATC	1327–1343 on COI
R3	CCAAAGAATCAAAATAAATGTTG	688–710 on COI
